# Thermally-responsive, nonflammable phosphonium ionic liquid electrolytes for lithium metal batteries: operating at 100 degrees celsius†
†Electronic supplementary information (ESI) available: Detailed ionic liquids synthesis, characterization, conductivity, cyclic voltammetry, battery cycling and those of other compositions; SEM images; energy density calculation. See DOI: 10.1039/c5sc01518a


**DOI:** 10.1039/c5sc01518a

**Published:** 2015-08-12

**Authors:** X. Lin, R. Kavian, Y. Lu, Q. Hu, Y. Shao-Horn, M. W. Grinstaff

**Affiliations:** a Departments of Chemistry and Biomedical Engineering , Boston University , Boston , MA 02215 , USA . Email: mgrin@bu.edu; b Department of Materials Science and Engineering , Massachusetts Institute of Technology , Cambridge , MA 02139 , USA

## Abstract

Rechargeable batteries such as Li ion/Li metal batteries are widely used in the electronics market but the chemical instability of the electrolyte limits their use in more demanding environmental conditions such as in automotive, oil exploration, or mining applications. In this study, a series of alkyl phosphonium ionic liquid electrolyte are described with high thermal stability and solubility for LiTFSI. A lithium metal battery (LMB) containing a tailored phosphonium ionic liquid/LiTFSI electrolyte operates at 100 °C with good specific capacities and cycling stability. Substantial capacity is maintained during 70 cycles or 30 days. Instant on-off battery operation is realized *via* the significant temperature dependence of the electrolyte material, demonstrating the robustness and potential for use at high temperature.

## Abstract 2

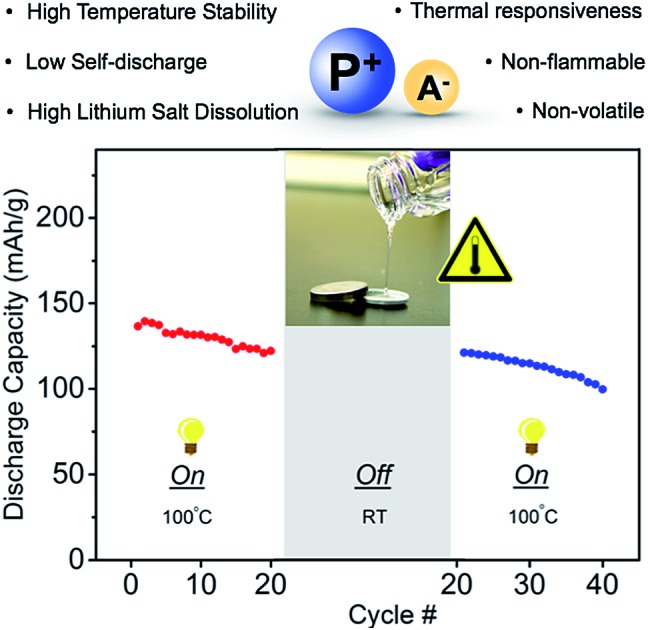
Lithium metal battery cycling at 100 °C is enabled by thermally-responsive, nonflammable phosphonium ionic liquid electrolytes.

## Introduction

Electrical energy storage (EES) devices that reliably and efficiently store, transport, and deliver energy are of key interest given the projected doubling of world energy consumption within the next several decades, combined with global efforts to reduce greenhouse gas emission.^1^,^2^ As the reliance on energy expands exponentially, high capacity batteries and supercapacitors are needed for use in consumer products as well as for use in industries such as oil exploration, mining, automotive, and military where demanding environmental conditions (especially high temperatures) are present.^3^ While a number of breakthroughs are reported describing new electrode materials with improved energy/power density,^4^–^13^ one limiting factor that precludes EES devices from practical use in the above-mentioned applications is the thermal stability of the electrolytes. Conventional electrolytes in batteries are carbonate based organic solvents (*e.g.*, ethylene carbonate, dimethyl carbonate) exhibiting low flash points and boiling points, which lead to severe safety issues for use in large-scale applications or at elevated temperatures.^14^,^15^

Non-flammable and non-volatile electrolytes, such as those based on ionic liquids (IL) and polymers, are being explored.^16^–^26^ For example, advances in solid polymer electrolytes (SPE) have lead to batteries that operate at elevated temperatures between ≈50 and 120 °C.^21^,^27^,^28^ However, these systems either showed limited cyclability (<30 cycles) with significantly decreased capacity, required very slow current rates, or demonstrated short cycle life at higher temperatures (>80 °C). Moreover, when stored at room temperature between operation at elevated temperatures, significant capacity loss can be observed.^29^ Although SPEs offer a number of important advantages, we are focused on ionic liquids as these materials usually possess higher ionic conductivity and better interfacial contact with the electrodes.^17^,^19^,^30^,^31^ Within this family of materials, imidazolium, piperidinium, and ammonium cations have been extensively studied for room temperature applications,^24^,^30^,^32^–^34^ however the investigation of their performance at elevated temperature is rare and satisfactory battery cycling above 60 °C has not been demonstrated.^29^,^35^,^36^ Phosphonium ionic liquids are less studied and exhibit both better chemical and thermal stability than imidazolium, pyrrolidinium or other nitrogen based ionic liquid cations, but with low conductivity at room temperature.^33^,^37^–^41^ We reasoned that their use at elevated temperatures would increase conductivity, *via* a reduction in viscosity, while maintaining superior thermal stability. In addition, the ionic liquid would serve as a media for ion transport at high temperatures but not at low temperatures, thus providing a reversible, thermally responsive on-off battery function.

Herein, we describe a lithium metal battery (LMB) that provides power for applications at 100 °C. Lithium metal was chosen as it is one of the most promising anode materials that can provide high theoretical capacity and high cell voltage. Specifically, we report: (1) the synthesis of a series of non-flammable, thermally stable phosphonium ionic liquid electrolytes and the subsequent identification of a lead candidate; (2) the significant temperature dependence on ion conductivity and viscosity of the phosphonium ionic liquids; (3) the dissolution of lithium bis(trifluoromethanesulfonyl)imide (LiTFSI) in the phosphonium electrolyte to give high (up to 1.6 M) concentrations; (4) the wide electrochemical stability window of the phosphonium electrolyte; (5) the successful performance of Li/phosphonium + LiTFSI electrolyte/LiCoO_2_ cells at 100 °C; and (6) the temperature dependent on-off battery operation enabling powering at 100 °C while remaining off between work transitions or when stored, thus, conserving overall battery lifetime.

## Results and discussion

**Fig. 1:**
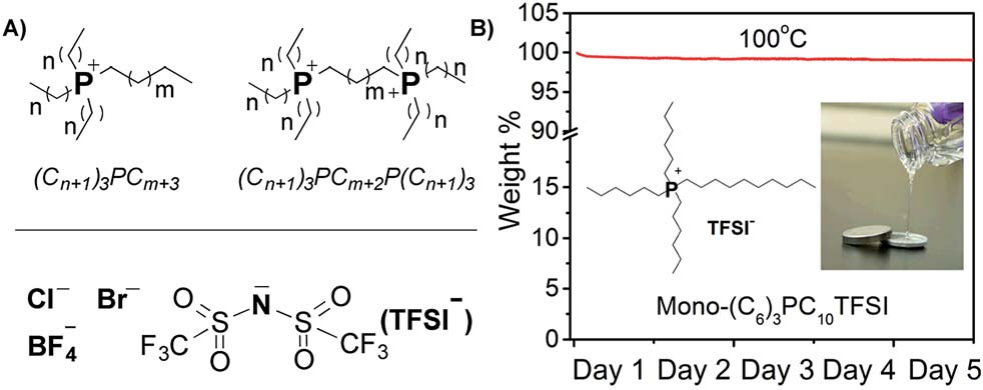
Chemical structures of ionic liquids under investigation. (A) Ionic liquids investigated in pre-screening; (B) picture and long-term thermal stability of the selected ionic liquid (mono-(C_6_)_3_PC_10_TFSI) for battery testing.

**Fig. 2:**
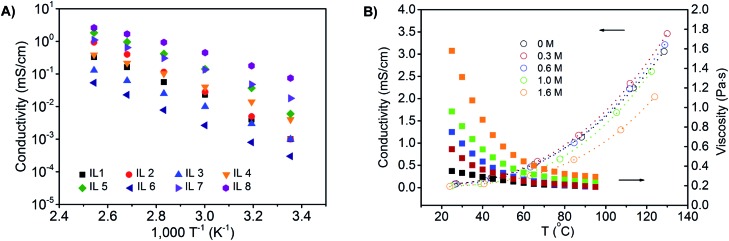
(A) Conductivity of a series of phosphonium ionic liquids with varied numbers of phosphonium centers, alkyl chain length and anions. IL 1: di-Cl(C_6_)_3_PC_10_P(C_6_)_3_Cl; IL 2: di-Cl(C_4_)_3_PC_10_P(C_4_)_3_Cl; IL 3: di-Cl(C_8_)_3_PC_10_P(C_8_)_3_Cl; IL 4: mono-(C_6_)_3_PC_10_Cl; IL 5: mono-(C_4_)_3_PC_6_Br; IL 6: di-Cl(C_8_)_3_PC_2_P(C_8_)_3_Cl; IL 7: mono-(C_6_)_3_PC_10_BF_4_; IL 8: mono-(C_6_)_3_PC_10_TFSI. (B) Conductivity and viscosity of mono-(C_6_)_3_PC_10_-TFSI loaded with different concentrations of LiTFSI as a function of temperature.

First, we synthesized a series of phosphonium ionic liquids that have different numbers of phosphonium cations (mono- and di-) and varied alkyl chain (C2, C6, and C10) lengths, and then paired them with different anions (Cl^–^, Br^–^, TFSI^–^, BF_4_^–^) to identify electrolyte compositions for battery use at elevated temperatures (Fig. 1; see ESI† for synthetic procedure details). From a design perspective, aliphatic alkyl chains, without allyl, hydroxyl, *etc.* reactive groups, are used to enhance chemical and electrochemical stability; alkyl chain asymmetry around the phosphonium is maintained to minimize potential crystallization or packing interactions; dicationic phosphoniums are evaluated given their enhanced thermal stability compared to mono-phosphoniums; counter ion size and basicity are altered to vary viscosity. Increasing the chain length from C2 to C10 enhances thermal and electrochemical stability while selection of the TFSI anion decreases the viscosity and increases the ion conductivity elative to the chloride, bromide and tetrafluoroborate samples. Phase transitions are observed for dicationic phosphoniums between –70 and 100 °C. The diphosphonium ionic liquids exhibit higher decomposition temperatures and viscosities, but lower conductivities than the corresponding monophosphoniums (see Table S1† and Fig. 2(A) for full characterization of screened ILs).

Based on this pre-screening, we selected the mono-(C_6_)_3_PC_10_-TFSI electrolyte composition because it possesses a high decomposition temperature (355 °C), no phase transitions, no thermal decomposition at 100 °C, and good conductivity at 100 °C (1.7 mS cm^–1^). When the electrolyte was exposed to an open flame for a couple of seconds, the electrolyte did not catch fire and is non-flammable (see photographs in Fig. S1†). Cyclic voltammetry using a three-electrode Li/Li/Pt setup, at 25 and 100 °C between –0.5 and 6.5 V (*vs.* Li^+^/Li) at 1 mV s^–1^, reveals that the composition is stable up to 5.0 V (see Fig. S2†). Above 5.0 V, TFSI anion degradation starts to occur, which is consistent with literature reports.^18^,^21^,^42^ The electrolyte mixture is cathodically stable until lithium plating and stripping occur around 0 V, respectively, during the initial cycles. At later cycles, the lithium plating and stripping peaks disappear suggesting a passive layer^43^ is formed between lithium metal and the ionic liquid electrolyte (see Fig. S2†).

LiTFSI can be dissolved in the mono-(C_6_)_3_PC_10_-TFSI up to a concentration of 1.6 M at 25 °C, and the ionic conductivities and viscosities for the mono-(C_6_)_3_PC_10_-TFSI with 0.3, 1.0, and 1.6 M LiTFSI as a function of temperature are shown in Fig. 2B. At room temperature, all of the compositions display relatively low conductivities (about 0.01 mS cm^–1^), whereas at 100 °C the conductivities increase by two orders of magnitude and are up to about 2 mS cm^–1^. Higher salt concentrations afford a more viscous mixture and less conductivity, while increasing the temperature significantly decreases the viscosity, and increases the conductivity.

**Fig. 3:**
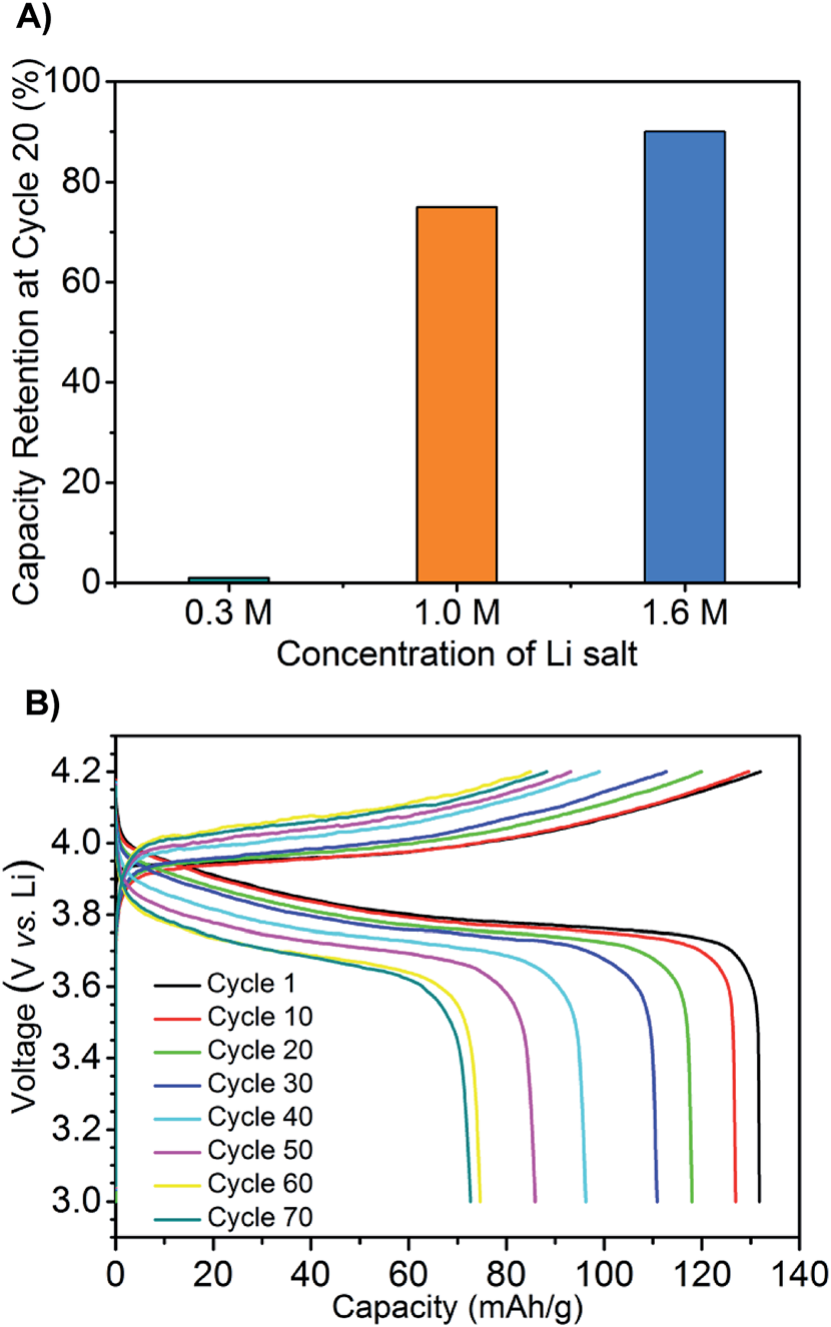
(A) The percent of specific capacity of cycle 1 at cycle 20 for 0.3 M, 1.0 M and 1.6 M compositions respectively. (B) Galvanostatic charge–discharge cycling for 1.6 M LiTFSI composition, current rate at C/7.

Next, prototype coin cells were assembled using a lithium metal negative electrode, a LiCoO_2_ positive electrode, and the mono-(C_6_)_3_PC_10_-TFSI with 0.3, 1.0, and 1.6 M LiTFSI. We recognize that the cathode material is not optimal for long term (months), high temperature applications, and this is a separate area of active investigation (*e.g.*, LiFePO_4_).^44^,^45^ Galvanostatic charge–discharge cycling from 3.0 V to 4.2 V, with a current rate at C/7, shows that battery performance is significantly influenced by LiTFSI concentration. All three batteries possessed high capacities of about 135 mA h^–1^ at the initial cycle similar to conventional room temperature LiCoO_2_ based batteries. However, the battery with 0.3 M LiTFSI suffered from significant capacity loss during cycling, which decreased to less than 5 mA g h^–1^ after 20 cycles along with low coulombic efficiency (Fig. 3A and S3†). In comparison, the batteries containing 1.0 and 1.6 M LiTFSI exhibited superior performance retaining 75 and 90% of the capacities after 20 cycles, respectively. High columbic efficiencies (>98%) are also reached during the first few cycles. Building on these encouraging results, a battery with the 1.6 M LiTFSI mono-(C_6_)_3_PC_10_-TFSI electrolyte was run for 30 days at 100 °C and cycled for 70 times (Fig. 3B). The battery had an initial high capacity of ∼135 mA h g^–1^ which decreased to 70 mA h g^–1^ after 70 cycles; this value still represents more than 50% of the initial capacity.

Given the above performance at 100 °C, the battery may be of potential use for powering a small sensor in the oil exploration/recovery industry. Efficient and safe recovery of petroleum from new and existing reservoirs requires diagnostic technologies for mapping the pH, sulfur content, temperature, pressure, *etc.* Current sensors are attached *via* wirelines for power, and, although useful, the areas that can be reached using wirelines are limited.^46^–^49^ The next generation of downhole oil sensors will be self-powered freely mobile devices that can migrate throughout a reservoir. The power requirements are estimated to be around 60 μW for these devices with micro-scale dimensions, and the battery must operate at elevated temperatures, such as 100 °C,^48^,^49^ for a short 1–2 day period (*e.g.*, temperature bore logger) or as long as 6 months.^46^ An energy density calculation reveals the first cycle provides 568 W h kg^–1^ (Fig. S4†), and this value decreases to 303 W h kg^–1^ after 70 cycles. Thus, the above LMB meets the power specifications and could power a microsensor for approximately 100 hours after one charge.

**Fig. 4:**
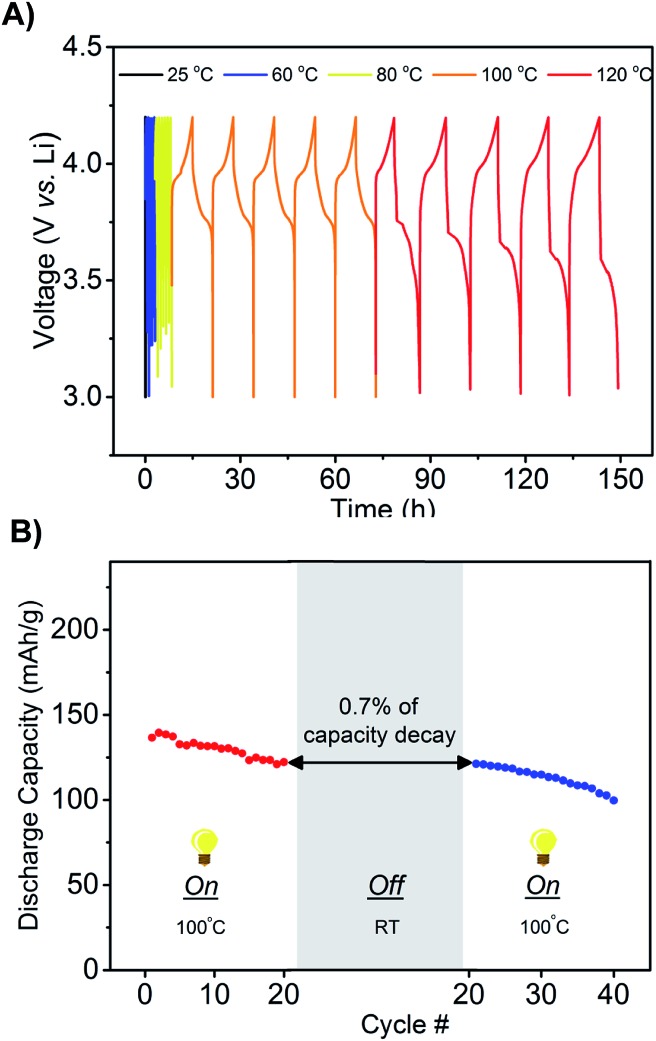
(A) Charge–discharge voltage profile of the thermally responsive ionic liquid electrolyte in a (Li, LiCoO_2_, mono-(C_6_)_3_PC_10_-TFSI, 1.6 M LiTFSI) cell (current rate C/7). Measurements are presented by heating from 25 to 120 °C and five cycles were measured at each temperature. (B) Demonstration of battery working only at high temperature and retain capacity at room temperature.

An additional design requirement for the battery is that it delivers power at elevated temperatures and remains off during work transitions or during storage at room temperature to conserve power. We hypothesized that the significant temperature-conductivity dependence of the phosphonium electrolyte would provide a mechanism for switching the battery between off and on as a function of temperature. Li ion transport would occur readily at high temperatures but not at low temperatures. Charge–discharge cycles were performed with the LMB (Li, LiCoO_2_, mono-(C_6_)_3_PC_10_-TFSI, 1.6 M LiTFSI) from 25 to 120 °C, and, as shown in Fig. 4A, the voltage–time profiles were measured for five cycles at each temperature. A constant current rate of C/7 was maintained to charge and discharge the battery between 3 and 4.2 V. At 25 °C, the battery does not operate due to a lack of ion mobility, which forces the voltage to quickly switch between the maximum and minimum. This result is in agreement with the conductivity measurements. Increasing the temperature to 60 or 80 °C decreases the viscosity but still afforded relatively fast charge and discharge cycles – characteristic of a viscous electrolyte with poor ion mobility-, and therefore low capacity. However, upon increasing the temperature to 100 °C, the battery functions with full charge and discharge cycles. Further increasing the temperature to 120 °C showed good capacity retention over 5 cycles, however the capacity decreased with additional cycles as the LiCoO_2_ cathode deteriorates at these high temperatures. A second experiment was performed to test the robustness of the battery's response towards temperature, as well as the ability to retain capacity after subjected to a significant temperature change. The LMB was operated at 100 °C for 20 charge–discharged cycles and then placed at rest for 2 weeks at room temperature. As shown in Fig. 4B, upon subsequent heating to 100 °C and performing charge–discharged cycles, the battery exhibited no significant loss of capacity (∼0.7%) due to self discharge at room temperature. Even if the battery was cycled at room temperature, the capacity decay is minimal due to the property of the ionic liquid chosen (see Fig. S5†).

Our observations of battery performance dependency on LiTFSI concentration are in agreement with recent reports.^50^,^51^ The improvement on the capacity retention by increasing the salt concentration to 1.6 M may be attributed to the formation of a passive layer and/or the alleviation of the lithium dendrite formation. Although we have a working prototype, relatively fast capacity loss occurs during cycling compared to conventional ambient temperature Li batteries. The chemical stability of the ionic liquid was confirmed by NMR and mass spectroscopy before and after high temperature cycling, and therefore the electrolyte decomposition is not a likely cause. Potential causes include deterioration of the SEI, dendrite formation, and instability of the LiCoO_2_ cathode over time.^52^–^54^ Experimentally, we observe a growing layer at the lithium metal as the battery cycles from 1 to 70 times at 100 °C by SEM (see Fig. S6†). The composition of this interface, the mechanism of its formation, and the influence of the interface thickening on the capacity fading, particularly at high temperature, will be the focus of a future study.^55^,^56^

## Conclusions

In conclusion, a thermally stable Li metal battery is reported for operation at 100 °C. The phosphonium ionic liquid/1.6 M LiTFSI electrolyte possesses an electrochemical stability window spanning 5 V *versus* Li^+^/Li at 25 °C and 100 °C. At room temperature, the electrolyte shows relatively low conductivity (∼0.01 mS cm^–1^), comparable to those of the solid polymer electrolyte.^16^ Whereas at 100 °C, the conductivity dramatically increases to >1 mS cm^–1^. The battery operates for more than 30 days (70 charge–discharge cycles) with sufficient specific energy and capacity retention for potential short-term downhole oil applications. The phosphonium ionic liquids are promising as thermally and electrochemically stable electrolytes. Continued research on thermally stable, high energy density devices (batteries, supercapacitors^57^) will meet the power demands for industrial applications where operation at 100 °C or higher are required. Moreover, this application specific technology/market pull^58^–^60^ from industry will spur the study of electrochemical processes at high temperatures, as well as innovations in new materials and energy device configurations for high energy output, efficient energy transfer, and conservation of power.

## Experimental

Synthesis of phosphonium ionic liquids: trihexylphosphine (8.3 g, 29 mmol) and 1-chlorodecane (5.22 g, 29.6 mmol) were mixed together and heated to 140 °C for 24 h to obtain mono-HexC10Cl. Next, the mixture was placed under vacuum at 140 °C to remove any volatile components. A clear colorless liquid was obtained in 99% yield. Mono-HexC10Cl (7.75 g, 16.74 mmol) and LiTFSI (6.25 g, 21.76 mmol) were mixed together in 20 mL DCM/H_2_O (1 : 1) solvents. The mixture was stirred at room temperature overnight and washed by 3 × 15 mL of water. 1 N AgNO_3_ solution was used to confirm the complete elimination of chloride anion. The organic layer was dried on anhydrous MgSO_4_ and the solvent was removed under reduced pressure. The reactions for other ionic liquids followed a similar procedure.

Thermal gravimetric Analysis (TGA) measurements were performed with TGA Q50. All samples were heated from 20 to 500 °C at a heating rate of 20 °C min^–1^. The decomposition point was marked as the 10% weight loss of the original sample weight. In addition, long-term thermal stability was determined. The phosphonium electrolyte was heated at 100 °C for 5 consecutive days, showing zero weight loss combined with no chemical changed indicating the suitable characteristics for a safe and stable electrolyte materials. Samples were also tested with Differential Scanning Calorimetry (DSC) at a heating rate of 20 °C min^–1^ and a cooling rate of 10 °C min^–1^ from –70 to 200 °C. All samples were measured between 5 to 10 mg and scanned for three heat–cool cycles.

The conductivity measurements were performed using a Conductivity Meter (K912, Consort) that has a 4-electrode cell to prevent the polarization error and fouling of the electrode. The ionic liquid electrolytes were dried at 100 °C under high-vacuum overnight to remove any trace amount of moisture before testing. Samples were loaded in test tubes sealed with septum stopper in order to maintain N_2_ environment. A heating block was used to control the temperature and stirring was maintained during the measurement to maintain homogeneity. A 30 minute equilibration time was used at each temperature.

To analyze the electrochemical stability window lithium/lithium/platinum three-electrode system was assembled and sealed in the glove box. Then cyclic voltammetries at 1 mV s^–1^ between –0.2 V and 6.5 V *versus* Li^+^/Li were carried out using a Princeton Applied Research VersaStat. A multichannel Princeton Applied Research VersaStat battery tester was used for cycle testing. LiCoO_2_ discs (area = 1.4 cm^2^) were punched out of the electrode film (MTI), and assembled into CR2032 coin cells along with equal-sized metallic lithium discs (0.75 mm thick) and Celgard 480 separators. All cells were assembled in an argon-filled glove box with a dew point of –80 °C. The cells were equilibrated at 25 or 100 °C in an oven. The potential window was 3.0 V to 4.2 V at C/7.

Scanning electron microscopy (SEM) was performed using a Zeiss SUPRA 40VP field emission SEM. Samples were gently washed with dimethyl carbonate and were allowed to dry for 24 h in the glove box. The samples were loaded with the protection of argon and were imaged at an accelerating voltage of 1.5 to 2 kV.

## Supplementary Material

Supplementary information
