# Supplementary material for: Thermally-responsive, nonflammable phosphonium ionic liquid electrolytes for lithium metal batteries: operating at 100 degrees celsius
Source: Chem Sci. 2015 Aug 12;6(11):6601–6. doi: 10.1039/c5sc01518a (PMC5506629; doi:10.1039/c5sc01518a)
Supplement: Supplementary file 1 [file SC-006-C5SC01518A-s001.pdf]

Electronic Supplementary Material (ESI) for Chemical Science.
This journal is © The Royal Society of Chemistry 2015

Supporting Information

Thermally-responsive, Nonflammable Phosphonium Ionic Liquid Electrolytes for Lithium Metal Batteries: Operating at 100 Degrees Celsius

Xinrong Lin[a], Reza Kavian[b], Yi-Chun Lu[b], Qichao Hu[b], Yang Shao-Horn[b], and Mark W. Grinstaff*[a]

[a]Departments of Chemistry and Biomedical Engineering, Boston University, Boston, MA 02215.

[b]Department of Materials Science and Engineering, Massachusetts Institute of Technology, Cambridge, MA 02139

General and Materials

All chemicals were purchased from Aldrich or Acros at highest purity grade and used without further purification. All reactions were performed under nitrogen atmosphere. ^{1}H (400 or 500 MHz), ^{13}C (101 or 126 MHz) and ^{31}P (161 MHz) NMR spectra were recorded on Varian INOVA spectrometers. Electrospray mass spectra were obtained on an Agilent 1100 LC/MSD Trap with ESI and APCI sources.

Preparation of Mono-phosphoniums

Trihexylphosphine (8.3 g, 29 mmol) and 1-chlorodecane (5.22 g, 29.6 mmol) were mixed together and heated to 140 ^{o}C for 24 h to obtain mono-HexC10Cl. Next, the mixture was placed under vacuum at 140 ^{o}C to remove any volatile components. A clear colorless liquid was obtained in 99% yield. ^{1}H NMR ($CDCl_3$): δ0.85-0.94 (t, 12, CH_3); 1.21-1.38 (br, 24, CH_2); 1.43-1.59 (br, 16, CH_2-CH_2-P); 2.41-2.50 (br, 8, CH_2-P). ^{13}C NMR ($CDCl_3$): δ 13.71 and 13.87 (CH_3); 19.27 and 18.80 (CH_2-CH_3); 21-22 (CH_2); 28.75-

31.60 (CH_2-P). ^{31}P NMR ($CDCl_3$): δ35.46 (P^+). HR MS (ESI): $[M-Cl]^+$ found 427.4431 (theory: 427.4427). Elemental analysis: found: C, 72.53; H, 13.18 (theory: C, 72.60; H, 13.20).

Anion Exchange Reactions

Mono-HexC10Cl (7.75 g, 16.74 mmol) and LiTFSI (6.25 g, 21.76 mmol) were mixed together in 20 mL DCM/H_2O (1: 1) solvents. The mixture was stirred at room temperature overnight and washed by 3 × 15 mL of water. 1 N $AgNO_3$ solution was used to confirm the complete elimination of chloride anion. The organic layer was dried on anhydrous $MgSO_4$ and the solvent was removed under reduced pressure. The anion exchange reaction for other anions followed a similar procedure.

General Procedure for Thermal Measurements

Thermalgravimetric Analysis (TGA) measurements were performed with TGA Q50. All samples were heated from 20 to 500 ^oC at a heating rate of 20 ^oC/min. The decomposition point was marked as the 10% weight loss of the original sample weight. In addition, long-term thermal stability was determined. The phosphonium electrolyte was heated at 100 °C for 5 consecutive days, showing zero weight loss combined with no chemical changed indicating the suitable characteristics for a safe and stable electrolyte materials.

Samples were also tested with Differential Scanning Calorimetry (DSC) at a heating rate of 20 ^oC /min and a cooling rate of 10 ^oC /min from -70 to 200 ^oC. All samples were measured between 5 to 10 mg and scanned for three heat-cool cycles.

Conductivity Measurement

The conductivity measurements were performed using a Conductivity Meter (K912, Consort) that has a 4-electrode cell to prevent the polarization error and fouling of the electrode. The ionic liquid electrolytes were dried at 100 ^oC under high-vacuum

overnight to remove any trace amount of moisture before testing. Samples were loaded in test tubes sealed with septum stopper in order to maintain N_2 environment. A heating block was used to control the temperature and stirring was maintained during the measurement to maintain homogeneity. A 30-minute equilibration time was used at each temperature.

Table S1. Physicochemical properties of phosphonium ionic liquids under investigation and pre-screening for the electrolyte

Entry	Name	Mw (g/mol)	T_g[a] (°C)	T_m[a] (°C)	T_d[b] (°C)	Viscosity at 25 °C (1 Hz) (Pa.s)	Conductivity at 25 °C (uS/cm)	Conductivity at 100 °C (uS/cm)
1	Di-Cl$(C_6)_3PC_{10}P(C_6)_3$Cl	784.1	-30	60	385	2.6×10^5	1.3	162
2	Di-Cl$(C_4)_3PC_{10}P(C_4)_3$Cl	615.8	0	103	401	3.9×10^5	1.4	400
3	Di-Cl$(C_8)_3PC_{10}P(C_8)_3$Cl	952.4	N/A	-57	380	2.6×10^3	1.3	62
4	Mono-$(C6)_3PC_{10}$Cl	462.2	N/A	N/A	340	0.9×10^2	4.2	215
5	Mono-$(C4)_3PC_6$Br	366.4	-34	N/A	335	1.6×10^2	6.3	960
6	Di-Cl$(C_8)_3PC_2P(C_8)_3$Cl	840.2	-52	N/A	350	1.2×10^3	0.3	23
7	Mono-$(C_6)_3PC_{10}BF_4$	513.5	N/A	N/A	350	1.5	18.0	647
8	Mono-$(C_6)_3PC_{10}$TFSI	706.9	N/A	N/A	355	0.3	75.2	1690

[a] Determined from -70 to 200 °C. T_g or T_m missing in the table were not observed in the temperature range tested.

[b] The temperature at 10 % weight loss. Samples are protected by nitrogen during test.

General Procedure for Rheological Measurement

About 1 mL of each sample was placed on an AR 1000 Controlled Strain Rheometer from TA Instruments equipped with a peltier temperature control using a 20 mm diameter parallel aluminum plate. The gap was set to be 1.0 - 2.0 mm in all the runs. To minimize the effect of moisture in the air, the experiments were performed in a glove bag filled with nitrogen gas. Prior to each test, a pre-shear was done at shear rate 100 1/s for 10 s to

eliminate the physical memory of the sample, followed by a 15 minutes equilibrium step in order for the sample to reach a steady state condition.

Strain amplitude from 0.1 to 10 % was determined to lie within the linear viscoelastic region (LVR) via an oscillatory strain sweep at a fixed frequency (1 Hz). Dynamic shear measurements covering 0.628 - 628 rad/s were conducted to obtain dynamic viscosity, storage modulus (G'), loss modulus (G") and phase angle. Measurements were typically performed at 25 ^{o}C unless temperature effect was investigated. Oscillatory temperature sweep was conducted from 10 ^{o}C to 95 ^{o}C with increment of 5 ^{o}C and 1-minute equilibrium at each temperature. Strain and frequency were set to be 1.0 % and 1 Hz, respectively.

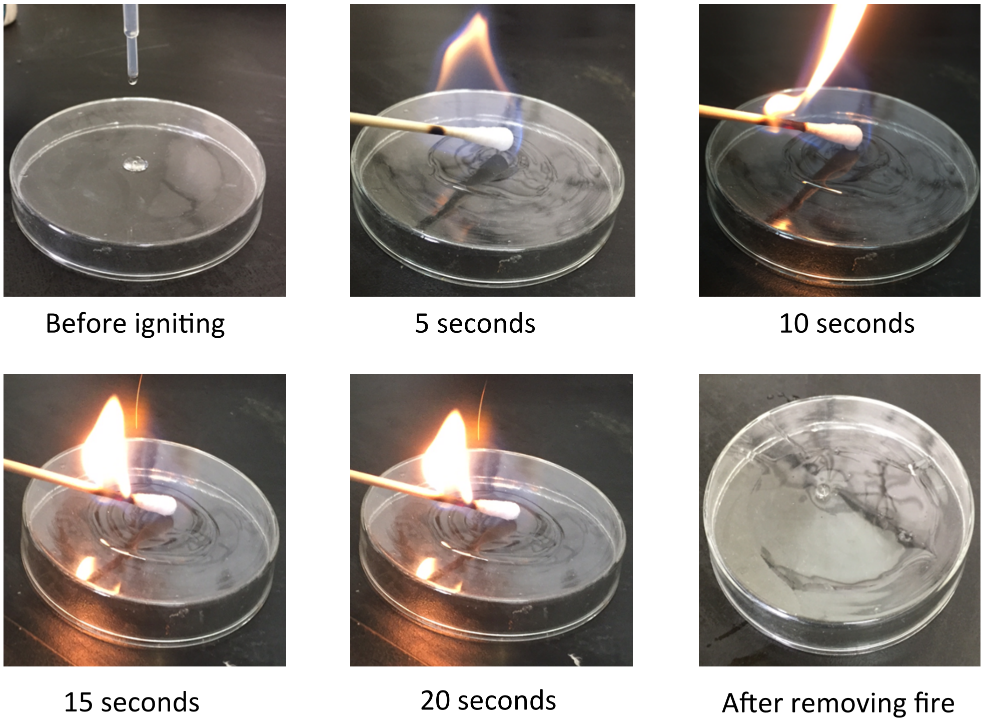

Figure S1. Photographs of the flammability experiment where the Mono-$(C_6)_3PC_{10}$TFSI ionic liquid is exposed to an open flame.

General Procedure for Cyclic Voltammetry

To analyze the electrochemical stability window lithium/lithium/platinum three-electrode system was assembled and sealed in the glove box. The experiments were carried out outside the glove box. The cells were equilibrated at 25 or 100 °C in an oven. Then cyclic voltammetries at 1 mV/s between −0.2 V and 6.5 V versus Li^+/Li were carried out using a Princeton Applied Research VersaStat.

General Procedure for Battery Cycling

A multichannel Princeton Applied Research VersaStat battery tester was used for

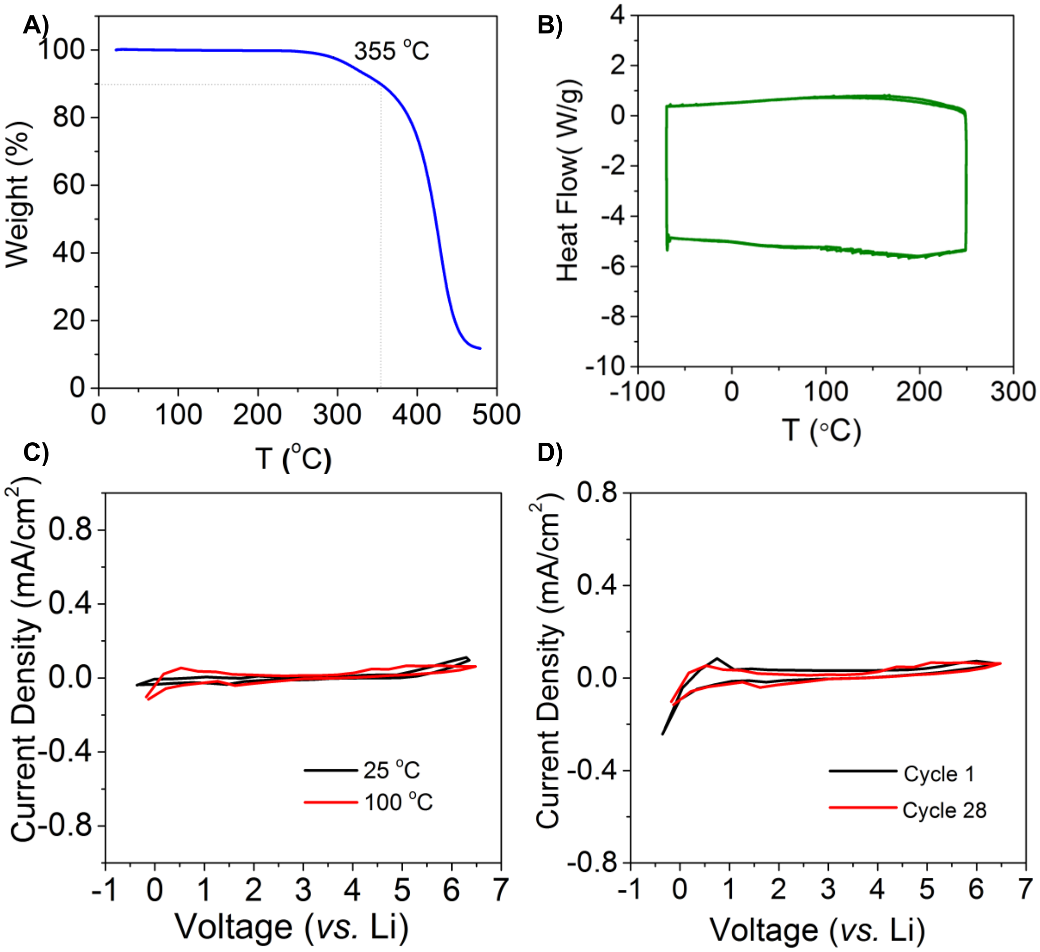

Figure S2. Thermal and electrochemical stability of Mono-$(C6)_3$PC10TFSI. A) Decomposition as a function of temperature by TGA; B) Demonstration of phase stability from -70 to 250 °C by DSC; C) Electrochemical window at 25 and 100 °C at cycle 28 at 1 mV/s; D) Electrochemical window at 100 °C at cycle 1 and 28 at 1 mV/s.

cycle testing. $LiCoO_2$ discs (area=1.4cm^2) were punched out of the electrode film (MTI), and assembled into CR2032 coin cells along with equal-sized metallic lithium discs (Sigma–Aldrich, 0.75 mm thick) and Celgard 480 separators. All cells were assembled in an argon-filled glove box with a dew point of −80 °C. The cells were equilibrated at 25 or 100 °C in an oven. Before high-temperature measurements were performed, each cell was first cycled (3 cycles) at room temperature at a small current (100uA) to form passivation layers on metallic lithium and to balance the cells. The potential window was 3.0 V to 4.2 V at C/7.

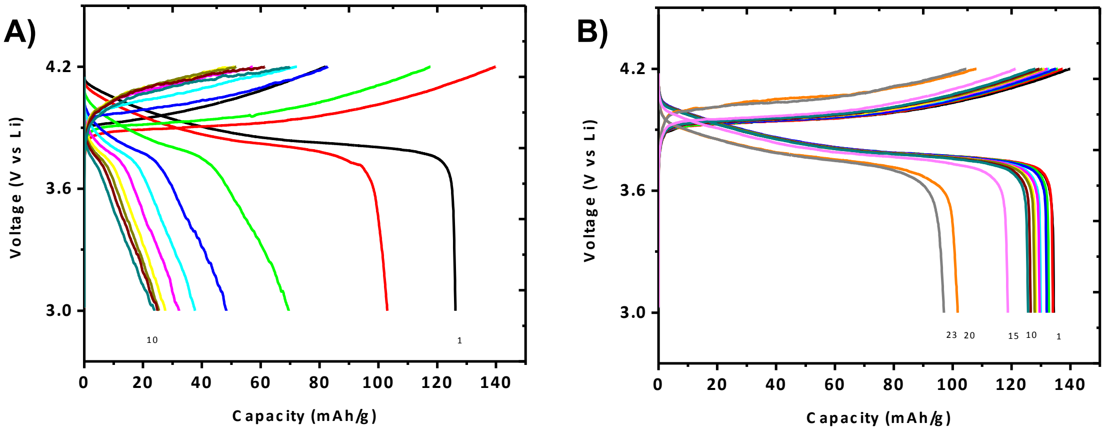

Figure S3. Galvanostatic charge-discharge cycling of 0.3M A) and 1.0M B) LiTFSI in Mono-$(C6)_3$PC10TFSI with a current rate at C/7 at 100 °C.

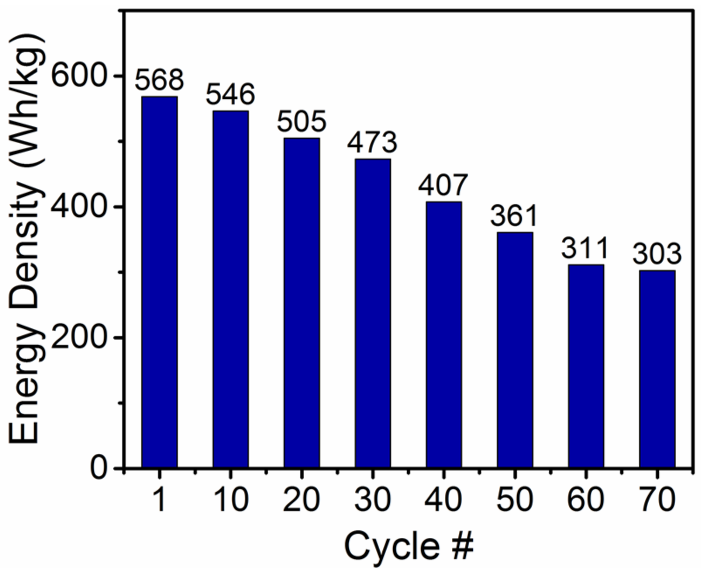

Figure S4. Energy density per electrode calculation at every 10 cycles for a prototype battery working at 100 ^{o}C

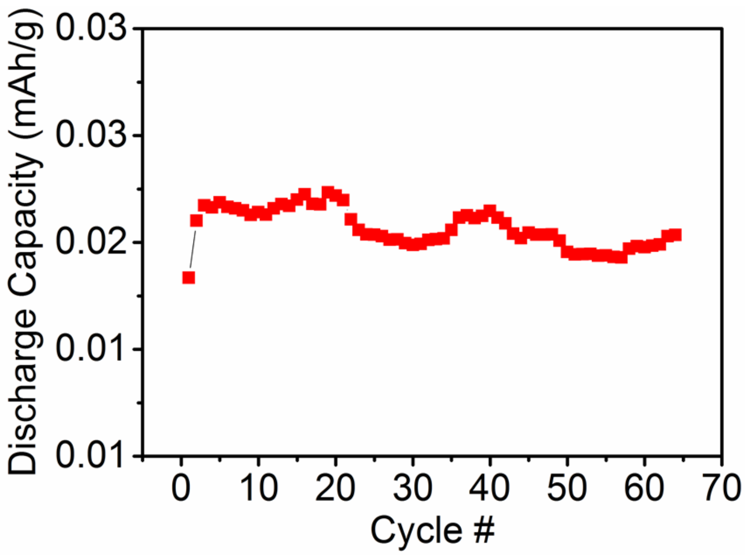

Figure S5. Galvanostatic charge-discharge cycling and minimum capacity decay of 1.6 M LiTFSI in Mono-$(C6)_3$PC10TFSI at room temperature with a current rate at C/7.

General Procedure for Scanning Electron Microscopy (SEM)

Scanning electron microscopy (SEM) was performed using a Zeiss SUPRA 40VP field emission SEM. Samples were gently washed with dimethyl carbonate and were allowed to dry for 24h in the glove box. The samples were loaded with the protection of argon and were imaged at an accelerat

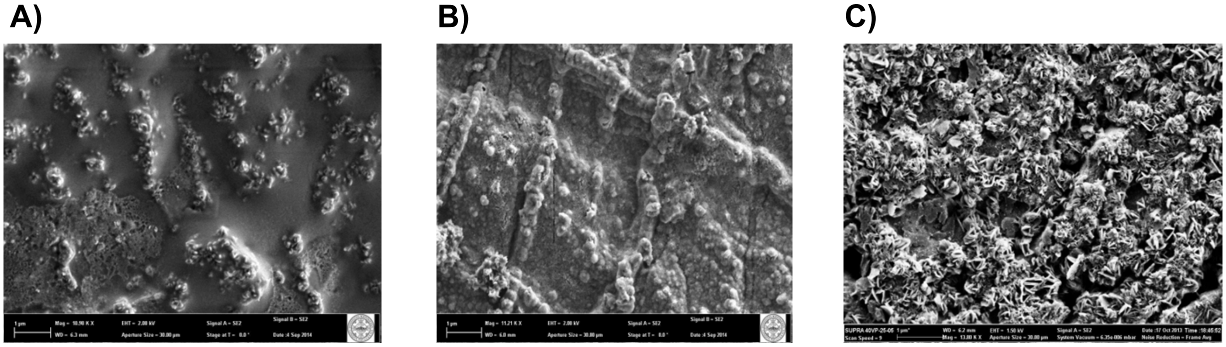

Figure S6. Surface growing at lithium anode at cycle 1 (left), 30 (middle) and 70 (right).
